# A single immunization with a modified vaccinia Ankara vectored vaccine producing Sudan virus-like particles protects from lethal infection

**DOI:** 10.1038/s41541-022-00512-x

**Published:** 2022-07-25

**Authors:** Delphine C. Malherbe, Arban Domi, Mary J. Hauser, Caroline Atyeo, Stephanie Fischinger, Matthew A. Hyde, Julie M. Williams, Galit Alter, Farshad Guirakhoo, Alexander Bukreyev

**Affiliations:** 1 Department of Pathology, University of Texas Medical Branch, Galveston, TX USA; 2 Galveston National Laboratory, Galveston, TX USA; 3 GeoVax Inc, Atlanta, GA USA; 4 Ragon Institute of MGH, MIT, and Harvard, Cambridge, MA USA; 5 Department of Microbiology & Immunology, University of Texas Medical Branch, Galveston, TX USA; 6 Present Address: Department of Microbiology, Immunology and Molecular Genetics, University of Kentucky, Lexington, KY USA; 7 Present Address: VAXXINITY, Dallas, TX USA

**Keywords:** Live attenuated vaccines, Viral vectors, Ebola virus

## Abstract

A new vectored vaccine MVA-VLP-SUDV was generated against Sudan ebolavirus (SUDV) combining the advantages of the immunogenicity of a live attenuated vaccine vector (Modified Vaccinia Ankara, MVA) with the authentic conformation of virus-like particles (VLPs). The vaccine expresses minimal components to generate self-assembling VLPs in the vaccinee: the envelope glycoprotein GP and the matrix protein VP40. Guinea pigs vaccinated with one dose of MVA-VLP-SUDV generated SUDV-specific binding and neutralizing antibody responses as well as Fc-mediated protective effects. These responses were boosted by a second vaccine dose. All vaccinated animals which received either one or two vaccine doses were protected from death and disease symptoms following challenge with a lethal dose of SUDV. These data demonstrate single dose protection and potency of the MVA-VLP platform for use in emergency situations to contain outbreaks.

## Introduction

Several filoviruses are responsible for fatal outbreaks of severe human diseases: Ebola virus (EBOV), Sudan virus (SUDV), Bundibugyo virus (BDBV) and Tai Forest virus (TAFV)^1^. Recently, two vaccines against the deadliest of these viruses, EBOV, were approved for human use: Ervebo VSV-based vaccine^2^, as well as Zabdeno (Ad26.ZEBOV) and Mvabea (MVA-BN-Filo) heterologous prime/boost vaccine^3^. Since its discovery in 1976, SUDV triggered eight outbreaks that infected 779 people and killed 412 people – 53% of infected people; the virus is responsible for the greatest number of ebolavirus outbreaks after EBOV^4^. The extremely high case fatality rate, the significant transmissibility of SUDV and its potential use as a biological warfare and bioterrorism weapon underscore the importance of countermeasures against this virus. As such, development of safe and efficacious vaccines is a high priority for the WHO and the U.S. Biomedical Advanced Research and Development Authority (BARDA) which goals include having a monovalent SUDV vaccine in the next decade^5^. However, there is currently no approved vaccine against SUDV, although several vaccine candidates were demonstrated to protect laboratory animals against the disease in preclinical studies^6–10^.

Modified Vaccinia Ankara (MVA) is well suited as a viral vaccine platform because of its excellent safety profile, immunogenicity, thermostability, genome coding capacity for multi-antigen expression and the lack of impairment of protective immune response by pre-existing orthopoxvirus-specific immunity^11^. Our Modified Vaccinia Ankara virus-like particle (MVA-VLP) vaccine platform was used to develop the vaccine candidate MVA-VLP-SUDV, which expresses the minimal components required for self-assembling filovirus VLPs: the glycoprotein GP and the matrix protein VP40^12^. MVA-VLP-SUDV combines the advantages of the authentic conformation of VLPs with the immunogenicity and safety of a live, attenuated virus vector. Previously the vaccine platform was used to develop protective vaccines against EBOV^12^ and Marburg virus^13^. Here we show that a single dose of MVA-VLP-SUDV is immunogenic and fully protective against lethal challenge suggesting that it is an ideal candidate for use in outbreak settings.

## Results

### Design and development of MVA-VLP-SUDV

**Fig. 1:**
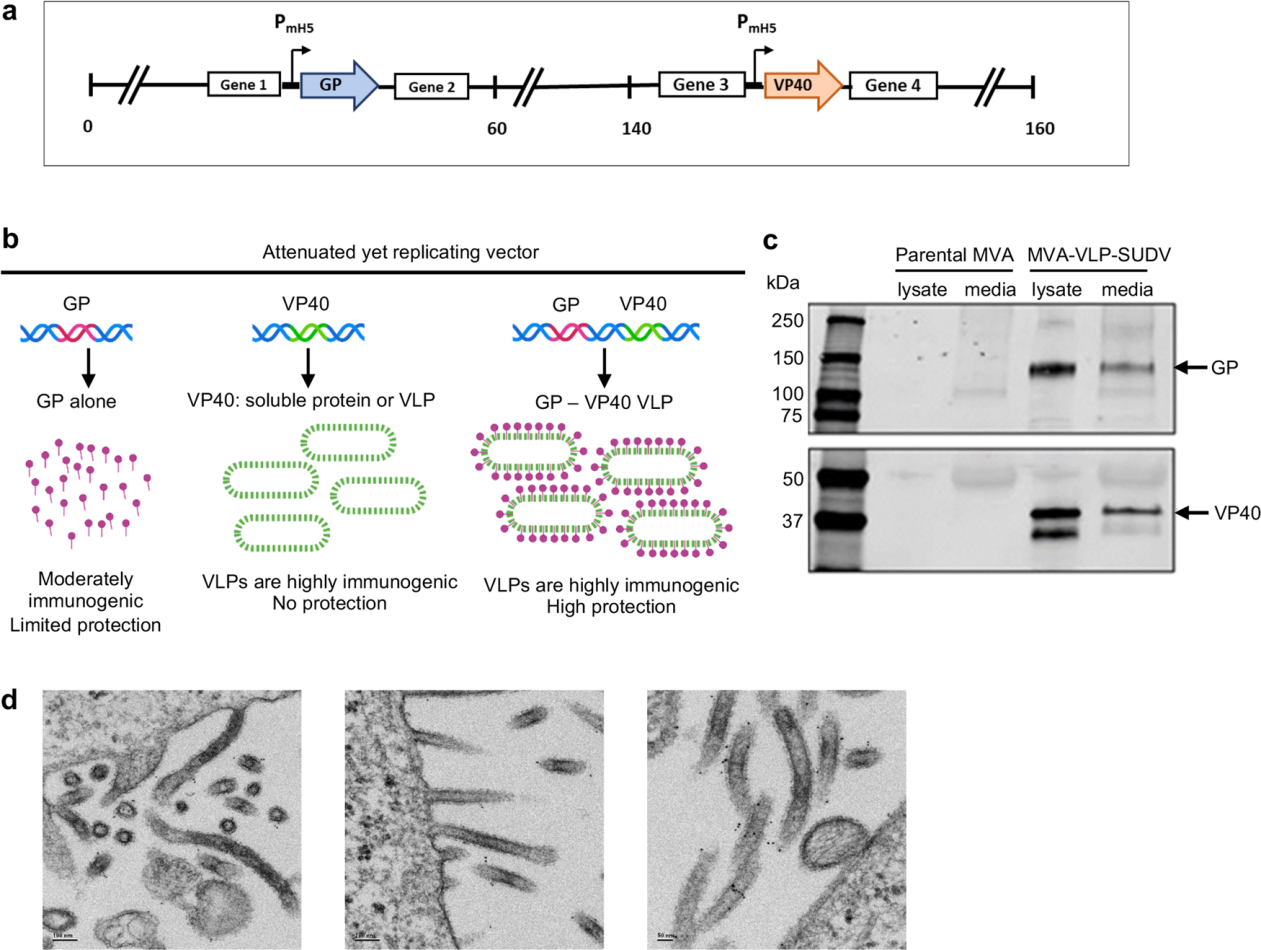
Design and characterization of MVA-VLP-SUDV vaccine. **a** MVA-VLP-SUDV construction. Sequences encoding SUDV GP and EBOV VP40 were inserted into the MVA backbone at two distinct sites between essential genes under direction of the modified H5 promoter. **b** Schematic representation of the mechanisms resulting in high immunogenicity of the MVA-VLP-SUDV construct. **c** MVA-VLP-SUDV antigen expression. Lysates and supernatant were collected from MVA-VLP-SUDV or parental MVA infected DF1 cells, subjected to Western blotting and probed with antibodies specific for SUDV GP (top) or VP40 (bottom). Expression of SUDV GP and EBOV VP40 was visualized in both the lysate and supernatant of recombinant vaccine infected cells. **d** VLP formation from MVA-VLP-SUDV infected cells. Thin layer sections of MVA-VLP-SUDV infected DF1 cell monolayers were visualized by TEM and reveal filamentous particle structure budding from the cell surface similar to native SUDV. Immunogold staining is specific for SUDV GP. Representative micrographs are shown with scale bars depicted on the bottom left corner. The scale bar sizes for the three microphotographs, from left to right are: 100 nm, 100 nm, 50 nm.

The MVA-VLP-SUDV vaccine construct was generated by insertion of SUDV GP isolate EboSud-603 2012 and VP40 EBOV isolate EBOV/H.sapiens-tc/GAB/1996/1kot sequences between MVA essential genes. We used VP40 of EBOV rather than SUDV because the VP40 protein by itself does not induce a virus-neutralizing antibody response, and a universal ebolavirus VLP-based MVA vector with pre-inserted EBOV VP40 transcriptional unit allows rapid development of a vaccine against any ebolavirus by insertion of a GP cDNA only. The filoviral sequences were codon-optimized for MVA use, and SUDV GP cDNA was introduced between two essential genes of the MVA genome (Fig. 1a, b). Western blot analysis confirmed protein expression of SUDV GP and EBOV VP40 in cells infected with the vaccine construct (Fig. 1c). SUDV antigens were detected in both intracellular cell lysate and culture supernatant. Self-assembly of VLPs in human HEK293 cells infected with MVA-VLP-SUDV for 24 h was confirmed by thin section transmission electron microscopy (Fig. 1d). Thus, MVA-VLP-SUDV infected cells produce budding SUDV VLPs with characteristic filamentous shape of filoviruses.

### Antibody responses to the MVA-VLP-SUDV vaccine

**Fig. 2:**
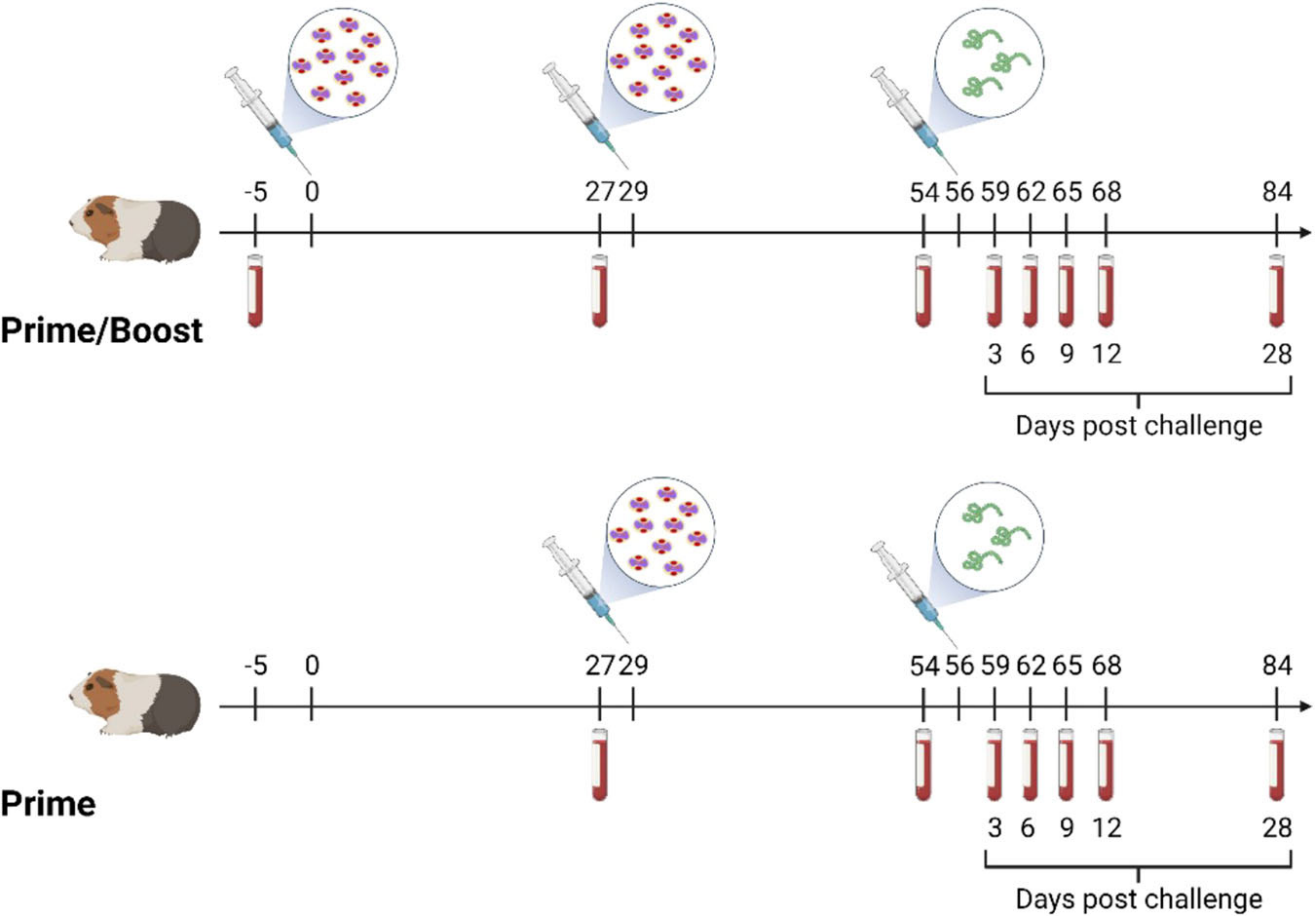
Study design. Dunkin-Hartley guinea pigs (*N* = 5 per group) were vaccinated with the MVA-SUDV vaccine IM either once (prime group) or twice (prime/boost group) at 29 days interval while the control group received PBS (not shown). Serum collected on days 27 and 54 was assessed for humoral responses. On day 56, all animals were challenged IP with guinea pig-adapted SUDV. Blood was collected on day 27 post vaccination and on the indicated days post challenge to assess humoral responses and vaccine efficacy. Figure partially created on BioRender.com.

**Fig. 3:**
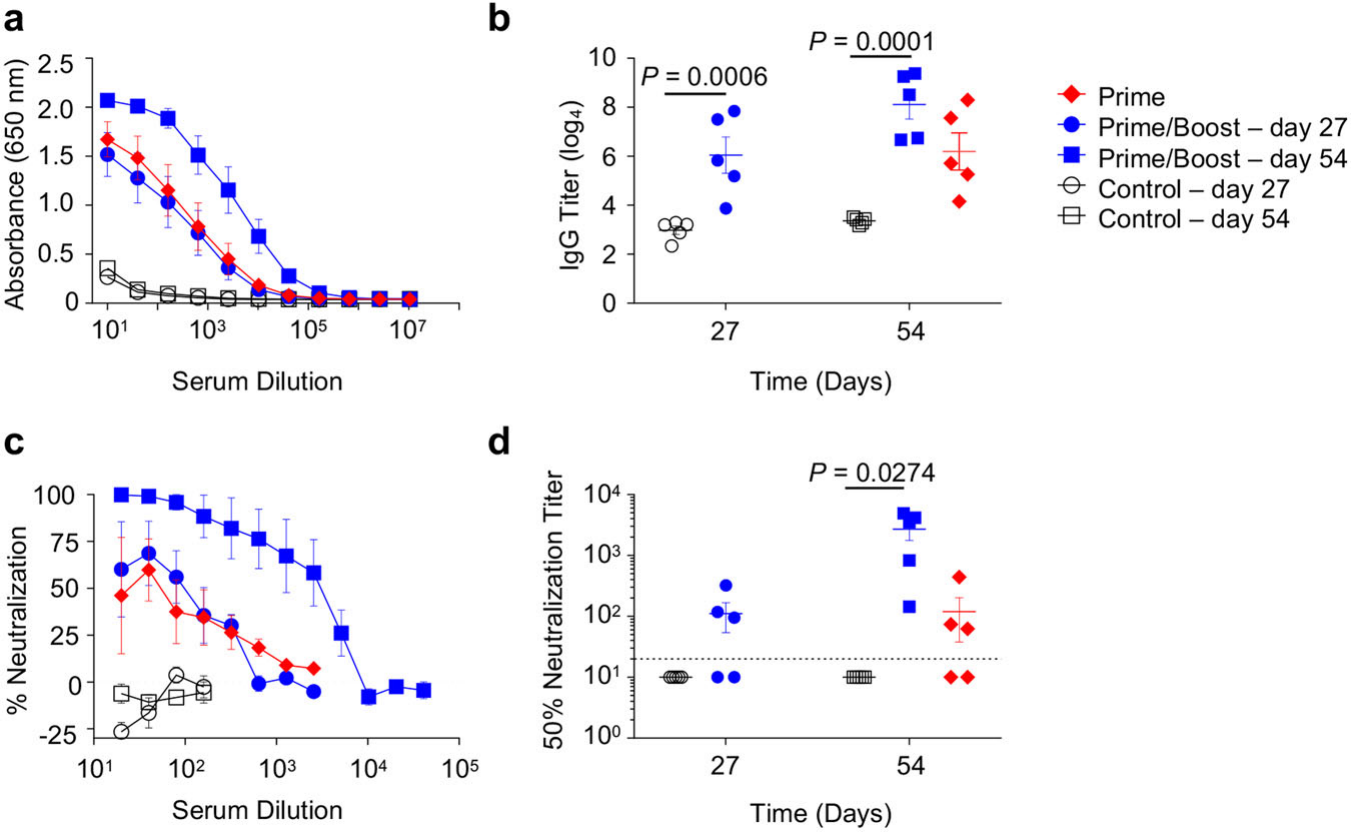
MVA-VLP-SUDV vaccine elicits SUDV-specific binding and neutralizing antibodies. Guinea pigs were vaccinated according the indicated regimens. Sera collected on days 27 and 54 were assessed for their ability to bind to SUDV GP (**a, b**) and neutralize SUDV Gulu (**c, d**). Mean values ± SEM. *N* = 5 animals per group. *P* values between prime/boost and control groups determined by 2-way ANOVA repeated measures followed by Bonferroni post-test.

The immunogenicity and efficacy of MVA-VLP-SUDV was evaluated in the guinea pig model in which one and two dose vaccine regimens were compared. Dunkin-Hartley guinea pigs (5 animals per group) were vaccinated twice intramuscularly (IM) with 10^8^ TCID_50_/animal of MVA-VLP-SUDV on days 0 and 29 in the prime/boost group, while the mock-vaccinated control group received saline solution. Guinea pigs in the prime group were vaccinated once intramuscularly with 10^8^ TCID_50_/animal of MVA-VLP-SUDV on day 29 (Fig. 2). On day 56, all guinea pigs were challenged intraperitoneally (IP) with a lethal dose of 10^3^ PFU guinea pig-adapted SUDV^14^. SUDV GP-specific IgG binding antibody titers were detectable after the first vaccine dose and were boosted by the second dose (Fig. 3a, b). Neutralizing antibody titers against SUDV strain Gulu were detectable after the first dose of MVA-VLP-SUDV and were boosted by the second dose (Fig. 3c); SUDV-neutralizing antibody responses were detected in 6 out of the 10 of vaccinated animals after one vaccination and in all vaccinated animals after the boost (Fig. 3d). The vaccine did not elicit cross-neutralizing antibodies against EBOV and BDBV (Supplementary Figure 1), a result that is not unexpected, as serum from SUDV human survivors did not neutralize EBOV^15^.

**Fig. 4:**
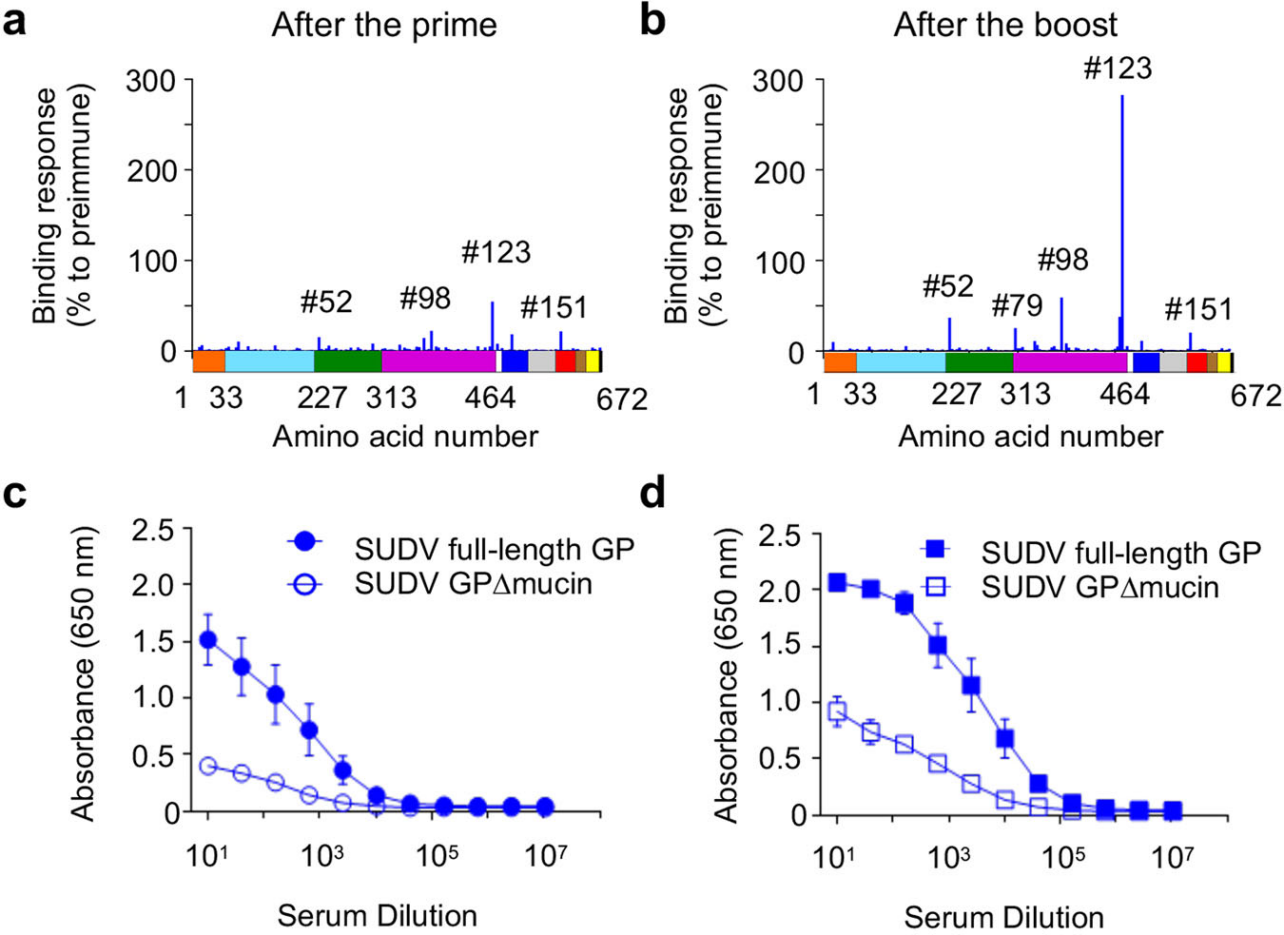
Amino acids 489-499 in GP1, by the furin cleavage site, are the main epitope targeted by MVA-VLP-SUDV. Sera collected after the first **a** and the second **b** vaccine doses were analyzed for binding to peptides matching the protein sequence of SUDV Gulu GP. Mean value of serum binding from three responders to each overlapping peptide is displayed as a percentage of the binding of pre-immune sera. The diagram below each graph shows the domains of GP in a linear fashion that correspond to the binding data: signal sequence (orange), receptor-binding domain (light blue), glycan cap (dark green), mucin-like domain (magenta), furin cleavage site (white), internal fusion loop (dark blue), heptad repeat 1 (light green), heptad repeat 2 (red), membrane proximal external region (brown), transmembrane domain (yellow) and cytoplasmic tail (black). Amino acid numbers for the N-terminus of the selected domains are indicated according ref. ^15^. Sera collected after the first vaccine dose (day 27) **c** and the second vaccine dose (day 54) **d** were tested by ELISA for binding to SUDV full-length GP and GP∆mucin. Mean values ± SEM.

To test antibody responses against linear epitopes, we used an array of 167 15-mer overlapping peptides which span the whole GP ectodomain of SUDV strain Gulu. The peptide array identified one main GP linear epitope at the C terminal end of GP1^16^(Fig. 4a, b), at amino acids 489–499, very close to the furin cleavage site^17^. In addition, we compared binding of antibodies in the immune sera to full-length SUDV GP versus the GP lacking the mucin-like domain (GP∆mucin). The mucin-like domain located at the C terminus of GP1 is one of the most dominant targets of the antibody response elicited by filovirus infections^18,19^. Our data show reduced but still detectable binding of antibodies to GP ∆mucin (Fig. 4c, d), suggesting immunogenicity of both the mucin-like domain and the other regions of GP in the vaccine construct.

### The MVA-VLP-SUDV vaccine elicits Fc-mediated protective responses

**Fig. 5:**
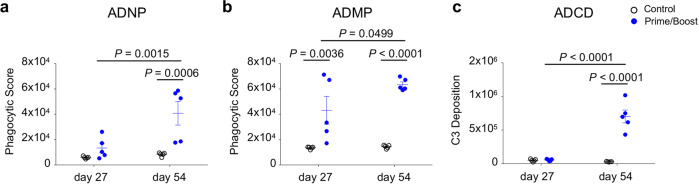
MVA-VLP-SUDV elicits antibodies capable of inducing multiple Fc-dependent innate immune effector functions. Sera collected at day 27 (after the first vaccine dose) and at day 54 (after the second vaccine dose) were assessed for Fc-dependent antibody functions when stimulated with SUDV GP protein. Guinea pig sera were incubated with GP coated fluorescent beads which were then incubated with either human neutrophils **a** or THP-1 monocytes **b**. Cells were then analyzed by flow cytometry to quantify phagocytic intake of fluorescent bead/antibody complexes. GP bound beads were incubated with guinea pig sera and exposed to guinea pig complement **c**; and C3 deposition (MFI of FITC) was measured by flow cytometry using a fluorescent anti-guinea pig C3 antibody. Mean values ± SEM. *N* = 5 animals per group. *P* values determined by 2-way ANOVA followed by Sidak’s multiple comparison test. *N* = 5 animals per group. ADNP, antibody-dependent neutrophil phagocytosis; ADMP, antibody-dependent monocyte phagocytosis; ADCD, antibody-dependent complement deposition.

Induction of Fc-mediated protective effects can play an essential role in vaccine-induced protection^20,21^. Thus, we investigated whether MVA-VLP-SUDV induces antibody-dependent neutrophil phagocytosis (ADNP) and antibody-dependent monocyte phagocytosis (ADMP) (Fig. 5a, b). In addition, we examined if the vaccine activates the complement system by assessing C3 deposition (ADCD, Fig. 5c). Gating strategies are displayed in Supplementary Figs. 2–4. For all three tested Fc-mediated functions, these responses were detected after the second dose (Fig. 5a–c). In addition, one vaccine dose was enough to elicit an ADMP response (Fig. 5b). Taken together, these results show the ability of the vaccine boost to induce diverse Fc-mediated protective functions by activating neutrophils, monocytes, and the complement system.

### A single dose of the MVA-VLP-SUDV vaccine protects animals from disease and death caused by SUDV

**Fig. 6:**
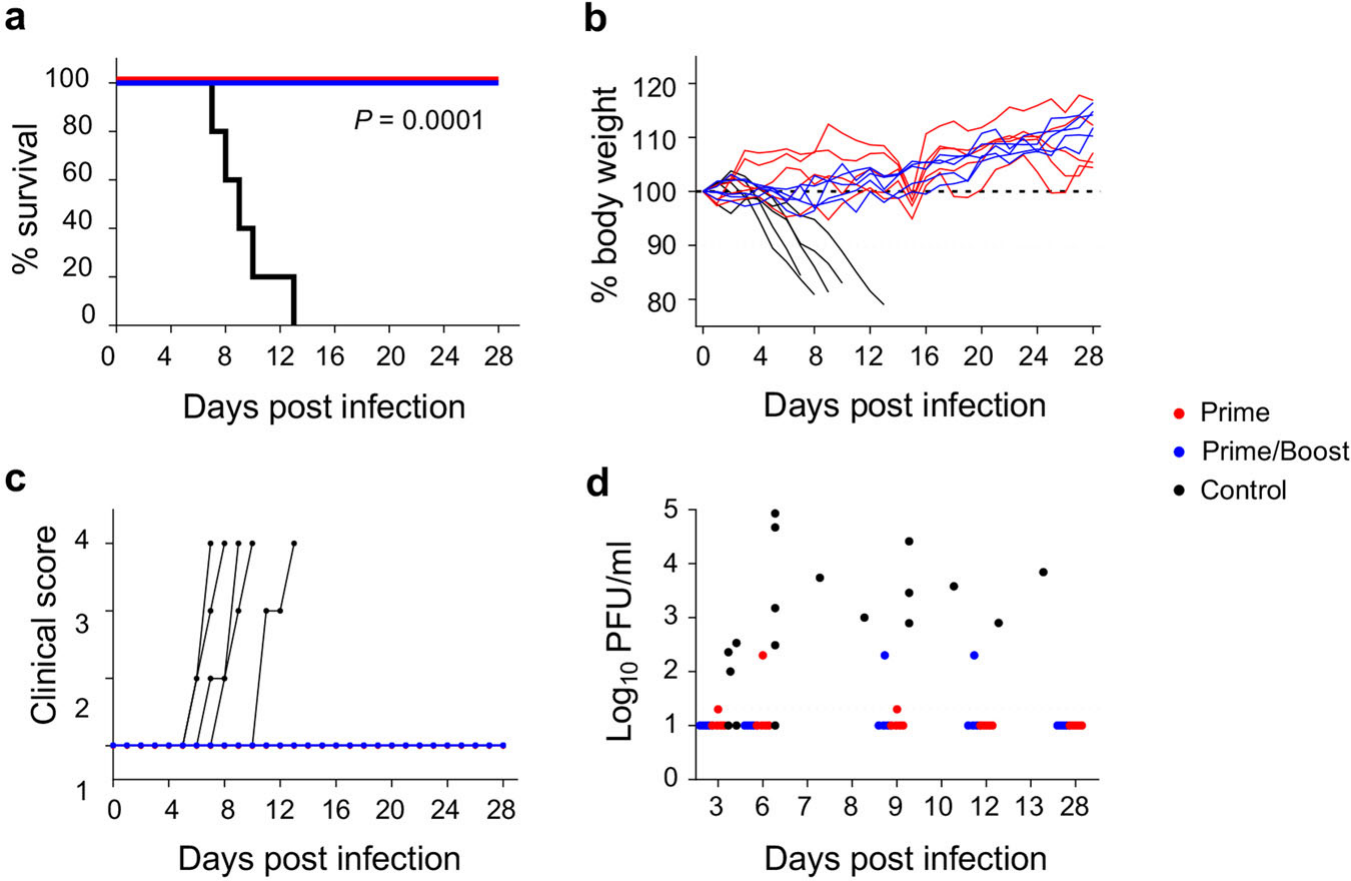
MVA-VLP-SUDV protects from death and disease caused by SUDV. Guinea pigs were vaccinated with the indicated vaccines: **a** survival, **b** weight loss, **c** disease scores, and **d** viremia following challenge with guinea pig-adapted SUDV are displayed. The prime group is in red, the prime/boost group in blue, and the control group in black. *N* = 5 animals per group. Survival: *P* = 0.0001 control animals versus vaccinated animals, Log-rank Mantel-Cox test.

Four weeks after the second or the single dose of the vaccine, animals were challenged with guinea pig-adapted SUDV strain Boneface^14^, which is heterologous to the vaccine GP component (Fig. 6a–d). In the control group, a significant loss of weight (*P* < 0.0001) was observed in all animals starting on day 4 (Fig. 6b), followed by other signs of disease starting on day 6 (Fig. 6c). Viremia was detected in all control animals (Fig. 6d) which reached moribund condition and were euthanized on day 7 through day 13 post challenge (Fig. 6a). In contrast, none of the vaccinated animals in both prime and prime-boost groups died or showed any loss of weight or other signs of the disease during the observation period. Three guinea pigs in the prime group and two guinea pigs in the prime/boost group developed very low transient viremia detected only at one timepoint per animal (Fig. 6d) but did not show any disease symptoms (Fig. 6c). Taken together, our data show that MVA-VLP-SUDV completely protected guinea pigs from death and disease caused by SUDV. Importantly there was no difference in outcomes between the prime and the prime/boost vaccine groups thus demonstrating the efficacy of one dose of the MVA-VLP-SUDV vaccine.

## Discussion

This study describes the development and testing of a novel vaccine candidate against SUDV. The MVA-VLP-SUDV vaccine platform is based on the MVA vector and expresses the minimal GP and VP40 genes to produce self-assembling VLPs in vivo thus combining the advantages of the live, attenuated and safe MVA vector and the authentic conformation of the GP antigen due to its incorporation in VLPs^12,13^ (Fig. 1b). The first vaccine dose elicited GP-binding and SUDV-neutralizing antibodies, which increased upon boosting. Following two vaccine doses, SUDV-specific neutralizing antibodies were detected in all vaccinated guinea pigs. Lazaro-Frias et al. also developed a MVA-based vaccine candidate expressing SUDV GP and SUDV VP40 and assessed it in mice immunized with a prime/boost strategy; while binding antibodies were elicited after two vaccine doses, neutralizing antibodies and protection from challenge were not assessed^22^. A peptide array revealed that MVA-VLP-SUDV induced antibodies that target linear epitopes which include amino acids 489-499 at the C terminus of GP1, adjacent to the furin cleavage site. Interestingly, immunization of mice with EBOV GP peptide 469-498 provided 100% protection from lethal EBOV challenge, and antibodies to this peptide were neutralizing^23^, so MVA-VLP-SUDV targets an epitope that overlaps with a known protective epitope against EBOV. Furthermore, another protective mechanism was at play for Marburg virus, which is another filovirus: murine mAb AGP127-8, whose epitope is also located at the C terminus of GP1 (Marburg virus GP amino acids 411-430), inhibited the release of Marburg viral particles from infected cells^24^. These data and our results show the potential protective functions of GP1 epitopes adjacent the furin cleavage site.

In addition to eliciting neutralizing antibodies, MVA-VLP-SUDV elicited Fc-mediated protective effects, including phagocytosis by monocytes and neutrophils, as well as activation of the complement system. Thus, our vaccine induces highly functional antibodies, capable of promoting distinct Fc-dependent innate immune effector functions by different immune cell types and of activating the complement system. These effector functions are likely to contribute to the protective efficacy provided by MVA-VLP-SUDV, since all animals in the prime group were protected from death and disease but only 60% had measurable neutralizing antibody titers. Therefore, we propose that the protective efficacy of MVA-VLP-SUDV likely results from both the direct neutralization by Fab domains and the Fc-mediated protective effects of vaccine-elicited antibodies.

All vaccinated guinea pigs survived the lethal SUDV challenge thus demonstrating the efficacy of the MVA-VLP-SUDV vaccine. Significantly, the vaccine protected from death and disease as no vaccinated animal lost weight or showed clinical symptoms. However, we detected low transient viremia in some vaccinated guinea pigs. Similar to our results, Warfield et al. detected transient low level viremia by plaque assay in 25% of NHPs vaccinated with three consecutive doses of conventional in vitro produced VLPs and challenged with a lethal dose of SUDV, and the virus was detected in 75% of vaccinated animals by qRT-PCR^6^. Importantly, guinea pigs that received one or two doses of MVA-VLP-SUDV were equally protected. The high protective efficacy of MVA-VLP-SUDV is a valuable feature for a vaccine deployed in emergency situations to contain viral outbreaks. Additional preclinical studies will characterize further the responses elicited by this vaccine candidate including SUDV-specific antibody isotypes and T cell responses as well as the duration of the protection and the orthopoxvirus-specific immune response and its effect on the primary and boost vaccinations. Moreover, in vitro testing will assess the absence of adventitious agents in the produced vaccine.

## Methods

### Generation and characterization of the MVA-VLP-SUDV vaccine

The MVA-SUDV-VLP vaccine construction was performed as previously described^12^ using SUDV isolate EboSud-603 2012 GP sequence (GenBank Accession number KC545390)^25^ and EBOV isolate EBOV/H.sapiens-tc/GAB/1996/1kot VP40 sequence (GenBank Accession number KC242798)^26^. The GP sequence was inserted between two essential vaccinia genes (I8R and G1L) using the pLW73 shuttle vector and the VP40 sequence was placed in a modified and restructured insertion site III using the pLW76 shuttle vector (Wyatt and Moss, unpublished data). To confirm expression of GP and VP40, DF1 cells infected with MVA-VLP-SUDV or the parental MVA (empty vector) at a MOI of 0.5 FFU/cell for 48 h at 37˚C. Then, cells were lysed, and the proteins were separated by SDS-PAGE under denaturing reducing conditions. Proteins were transferred to nitrocellulose, and the membrane was stained with anti-SUDV GP (1:1000 dilution, IBT cat # 0202-029) and anti-EBOV VP40 (1:1000 dilution, IBT cat # 0301-010) antibodies and visualized with the LI-COR Odyssey imaging system using AlexaFluor680 goat anti-rabbit IgG (Invitrogen cat # 21109) at a 1:10,000 dilution. To confirm the formation of VLPs by electron microscopy, HEK293 cells were infected with MVA-VLP-SUDV for 24 h, stained with rabbit anti-GP antibodies, fixed with 1% glutaraldehyde in 0.1 M phosphate buffer and incubated in 50 mM glycine to block residual aldehyde. Following incubation with goat anti-rabbit IgG secondary antibody conjugated to 6 nm gold particles (1:2,000 dilution, Aurion cat # 800.011), silver enhancement was done to increase the size of gold particles for subsequent viewing on a JEOL JEM-1400 electron microscope at The Robert P. Apkarian Integrated Microscopy Core of the Emory Integrated Core Facilities (EICF),

### Vaccination and SUDV challenge

This study was carried out in strict accordance with the recommendations described in the Guide for the Care and Use of Laboratory Animals of the National Research Council. UTMB is an AAALAC-accredited institution, and all animal work was approved by the UTMB Institutional Animal Care and Use Committee. All efforts were made to minimize animal suffering and all procedures involving potential pain were performed with the appropriate anesthetic or analgesic. The number of animals used in this study was scientifically justified based on statistical analyses of virological and immunological outcomes. Nine-week-old Dunkin-Hartley female guinea pigs (Charles Rivers Laboratories) were anesthetized with isoflurane for all procedures. On days 0 and 29, animals in the prime/boost vaccine group (*N* = 5) were inoculated with 10^8^ TCID_50_ in 100 µl injection volume via the intramuscular route, while animals in the control group (*N* = 5) were inoculated with 100 µl saline solution. On day 29, animals in the prime vaccine group (*N* = 5) were inoculated with 10^8^ TCID_50_ in 100 µl injection volume via the intramuscular route. Retro-orbital blood collections were performed on days -5 (five days prior the first immunization), 27 and 54. On day 56 vaccinated and control animals were exposed intraperitoneally to the targeted dose of 10^3^ PFU of guinea pig-adapted SUDV by the intraperitoneal route. Animals were monitored up to three times per day for weight loss and signs of disease and were euthanized when they reached the moribund state. The remaining animals were euthanized 28 days post infection. Retro-orbital blood collections were performed at 3, 6, 9, 12 and 28 days post challenge and at the time of euthanasia.

### Binding antibody response

Serum samples were tested for their ability to bind SUDV full length GP (IBT Bioservices, #0502-015) and SUDV ∆mucin GP missing amino acids 314-472 (IBT Bioservices, #0512-015) by ELISA^27^. Briefly, flat-bottom 96-well plates were coated overnight at room temperature with 8 ng per well of recombinant proteins diluted in phosphate buffered saline (PBS). Plates were washed five times in PBS with 0.1% Tween 20 and blocked with 3% dry milk in PBS for 1 h at 37 °C. After blocking, four-fold dilutions of guinea pig sera were prepared in blocking buffer and incubated on the GP coated plates for 1 h at 37 °C. Plates were washed as above and incubated with a 1:5,000 dilution of horseradish peroxidase-conjugated goat anti-guinea pig antibody (Jackson ImmunoResearch cat#106-035-003) for 1 h at 37 °C. Plates were washed as above, developed using the SureBlue ELISA substrate system (SeraCare), and color intensity was measured at 630 nm on BioTek Synergy HT microplate reader.

### SUDV GP peptide microarray

167 15-residue peptides overlapping by 4 amino acids were designed to cover the sequence of SUDV GP (Sudan virus/H.sapiens-tc/UGA/2000/Gulu-200011676) sequence. Peptides were synthesized and immobilized on glass slides by JPT Peptide Technologies. Each of the peptides was spotted via its N-terminus in triplicates to form one block of spots, and 21 blocks were spotted onto a slide. Dilutions 1:200 of serum samples (3 animals per timepoint day 0, 28 and 56) were incubated for 1 h at 30 °C followed by 4 washes in JPT Washing Buffer (1x TBS-Buffer (20 mM Tris, 136 mM NaCl, pH 7.4) + 0.1% Tween 20 (TBS-T)). Blocks were then incubated in 0.1 µg/ml anti guinea pig-Cy5 conjugated antibodies (Jackson ImmunoResearch cat # 706-175-148) followed by four washes. After an additional rinse in deionized water, the slide was dried by centrifugation. Fluorescent readings were recorded for each spot on the GenePix 4000b system (Molecular Devices) and analyzed with GenePix Pro 7 software (Molecular Devices).

### Neutralizing antibody response

Guinea pig sera were tested for neutralizing capabilities against SUDV strain Gulu as well as EBOV strain Mayinga and BDBV strain Uganda. Briefly, serum samples were heat-inactivated for 30 minutes at 56 °C and diluted in a 2-fold serial fashion in MEM (Gibco) with HEPES (Corning), gentamicin sulfate (Cellgro) and 10% guinea pig complement (MP Biomedicals). Fifty µl of each serum dilution was mixed with 200 PFU of virus in 50 µl. The serum/virus mixtures were incubated for 1 h at 37 °C. Fifty µl of the serum/virus mixtures were then transferred to Vero E6 cell monolayers in flat-bottom 96-well plates and incubated for 1 h at 37 °C. The serum/virus mixture was then removed and replaced with 1:1 overlay composed of 1% methylcellulose (Fisher Chemical) and 2X MEM (Gibco) supplemented with gentamicin sulfate (Corning) and 4% FBS (Gibco). Plates were incubated 5 days at 37 °C, then fixed with 10% Fisherbrand neutral buffered formalin (ThermoFisher Scientific) according to approved SOP and removed from biocontainment. Plates were washed 3 times with 1X DPBS (Corning) and blocked with 1X DPBS with 5% milk for 1 h. To visualize SUDV plaques, the monolayers were immunostained with 1 µg/mL human mAb BDBV43 (a gift from James Crowe, Vanderbilt University Medical Center) as primary antibody. Plates were washed three times in 1X DPBS before addition of HRP-conjugated goat anti-human IgG (SeraCare cat #5220-0330) at dilution 1:2,000 as secondary antibody. EBOV plaques were visualized with goat anti-EBOV serum at dilution 1:1,000 (gift of Thomas Ksiazek, UTMB) and HRP-conjugated bovine anti-goat IgG at dilution 1:2,000 (Jackson ImmunoResearch, cat #805-035-180). BDBV plaques were stained with 1 µg/mL human mAb BDBV52 (a gift from James Crowe, Vanderbilt) and HRP-conjugated goat anti-human IgG at dilution 1:2,000 (SeraCare, cat #5220-0330). Primary and secondary antibodies were diluted in blotto (5% solution of nonfat powdered milk in PBS). Finally, plates were washed three times in 1X DPBS (Corning), and plaques were visualized by incubation with AEC substrate (enQuire Bioreagents) at 37 °C for 30 min.

### Antibody-mediated neutrophil phagocytosis

Recombinant SUDV GP was biotinylated and coupled to 1 µm FITC^+^ Neutravidin beads (Life Technologies). Briefly, SUDV GP was biotinylated using Sulfo-NHS-LC-LC biotin (ThermoFisher Scientific), and excess biotin was removed using a Zeba desalting column (ThermoFisher Scientific). Biotinylated SUDV GP was incubated with Neutravidin-coated fluorescent beads (ThermoFisher Scientific) at 4 °C overnight (1 µg biotinylated protein per 1 µl of beads). Beads were washed twice with 0.1% BSA in PBS and resuspended in 100 µl of 0.1% BSA in PBS per 1 µl of coupled beads. Sera from vaccinated guinea pigs were diluted 1:100 in cell culture medium and incubated with GP-coated beads for 2 h at 37 °C. Freshly isolated neutrophils from donor blood were added at a concentration of 5.0 ×10^4^ cells per well and incubated for 1 h at 37 °C. Cells were stained at 1:100 with CD66b (Pacific Blue, Clone G10F5; Biolegend cat # 305111), fixed with 4% paraformaldehyde, and analyzed by flow cytometry on a Sartorius iQue flow cytometer, and a minimum of 30,000 events were recorded and analyzed. Neutrophils were defined as SSC-A^high^ CD66b^+^. The phagocytic score was determined using the following formula: (percentage of FITC^+^ cells) x (median fluorescent intensity (MFI) of the FITC^+^ cells)/10,000.

### Antibody-mediated cellular phagocytosis by human monocytes

Recombinant SUDV GP was biotinylated and coupled to 1 µm FITC^+^ Neutravidin beads. Serum samples from vaccinated guinea pigs were diluted 1:500 in culture medium and incubated with GP-coated beads for 2 h at 37 °C followed by addition of cells for 18 h. Cells were fixed with 4% paraformaldehyde and analyzed on a Sartorius iQue flow cytometer, and a minimum of 10,000 events were recorded and analyzed. The phagocytic score was determined as described above.

### Antibody-mediated complement deposition

Recombinant SUDV GP was biotinylated and coupled to 1 µm red fluorescent Neutravidin beads. Serum from vaccinated guinea pigs were diluted 1:10, 1:100, and 1:200 in culture medium and incubated with GP-coated beads for 2 h at 37 °C followed by the addition of reconstituted guinea pig complement (Cedarlane Labs) diluted in gelatin veronal buffer containing magnesium and calcium (Boston Bioproducts) and incubation for 20 min at 37 °C. Beads were washed twice with phosphate buffered saline containing 15 mM EDTA, and stained with an anti-guinea pig C3 antibody conjugated to FITC (1:100, MP Biomedicals cat #0855385). C3 deposition onto beads was analyzed on a Sartorius iQue flow cytometer, and a minimum of 10,000 events were recorded and analyzed. C3 deposition was calculated according to the following formula: (geometric mean fluorescent intensity of FITC of all beads) x (% of FITC + beads)/10,000.

### Analysis of viremia

Viremia was determined by titrating serum samples obtained after SUDV challenge by plaque assay on Vero E6 cells. Serum samples were serially diluted in a 1:10 series in 1X MEM (Gibco) supplemented with gentamicin sulfate (Corning) and 2% FBS (Gibco). Fifty µl of the serial dilutions were then transferred to Vero E6 cell monolayers in flat-bottom 96-well plates and incubated for 1 h at 37 °C. The dilutions were then removed and replaced with 1:1 overlay composed of 1% methylcellulose (Fisher Chemical) and 2X MEM (Gibco) supplemented with gentamicin sulfate (Corning) and 4% FBS (Gibco). Plates were incubated 5 days at 37 °C, then fixed with 10% neutral buffered formalin (Fisherbrand) according to approved SOP and removed from biocontainment. SUDV viral plaques were immunostained using the same method as described above for the neutralization assay.

### Biocontainment work

Work with infectious SUDV, EBOV and BDBV was performed in the BSL-4 facilities of the Galveston National Laboratory. Staff had the appropriate training and U.S. government permissions and registrations for work with filoviruses.

### Statistical analyses

2-way ANOVA followed by Bonferroni post-test; Kruskal-Wallis analysis followed by Dunn’s multiple comparison test and Log Rank (Mantel Cox) test were performed with GraphPad Prism for Windows (version 6.07). *P* < 0.05 was considered significant.

### Reporting summary

Further information on research design is available in the Nature Research Reporting Summary linked to this article.

## Supplementary information

Supplementary Figures 1, 2, 3, 4

REPORTING SUMMARY

## Data Availability

The datasets generated during and/or analyzed during the current study are available from the corresponding author on reasonable request.
