# Supplementary material for: A single immunization with a modified vaccinia Ankara vectored vaccine producing Sudan virus-like particles protects from lethal infection
Source: NPJ Vaccines. 2022 Jul 25;7:83. doi: 10.1038/s41541-022-00512-x (PMC9314403; doi:10.1038/s41541-022-00512-x)
Supplement: Supplementary file 1 — Supplementary Figures 1, 2, 3, 4 [file 41541_2022_512_MOESM1_ESM.pdf]

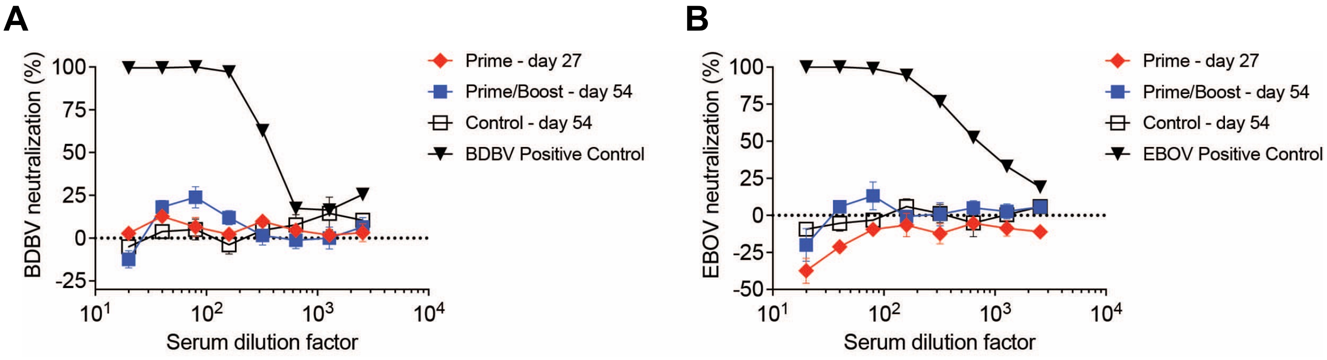


**Supplementary Figure 1. MVA-VLP-SUDV vaccine does not elicit neutralizing antibodies against BDBV and EBOV.** Guinea pigs were vaccinated according the indicated regimens. Sera were assessed for their ability to neutralize BDBV (A) and EBOV (B). Mean values ± SEM. N = 5 animals per group.

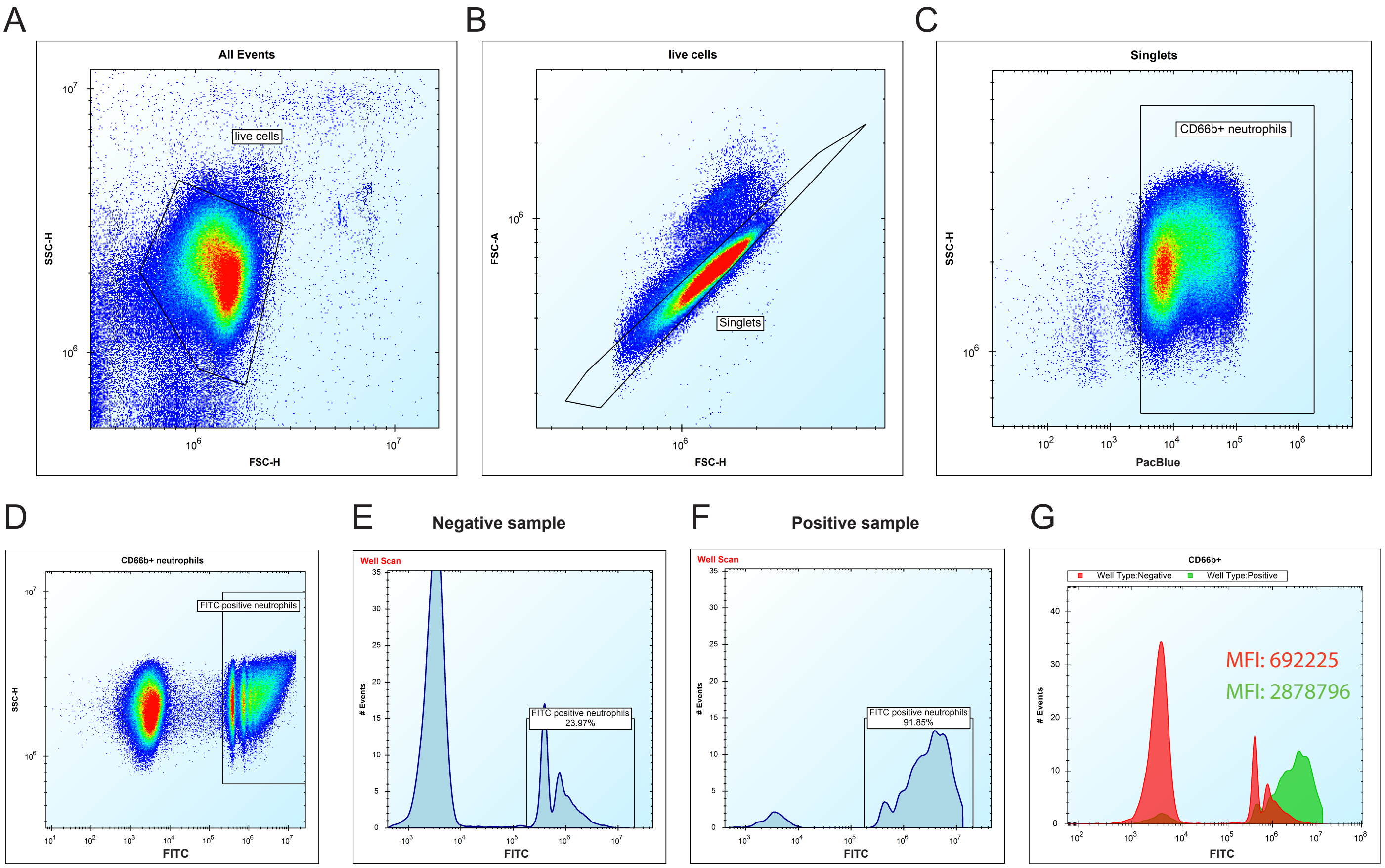


**Supplementary Figure 2. Gating strategy and positive/negative sample for ADNP.** (A) Gating strategy for gating on live cells. (B) Gating strategy for single cells. (C) Gating strategy for CD66+ neutrophils. (D) Gating strategy for gating on bead-FITC-positive cells. (E) Example of the percentage of neutrophils (CD66b+ cells) positive for beads for a negative sample. (F) Example of the percentage of neutrophils (CD66b+ cells) positive for beads for a positive sample. (G) Overlay of the fluorescence of neutrophils for a positive sample (green) and negative sample (red). The median fluorescence intensity (MFI) calculated for each sample is shown.

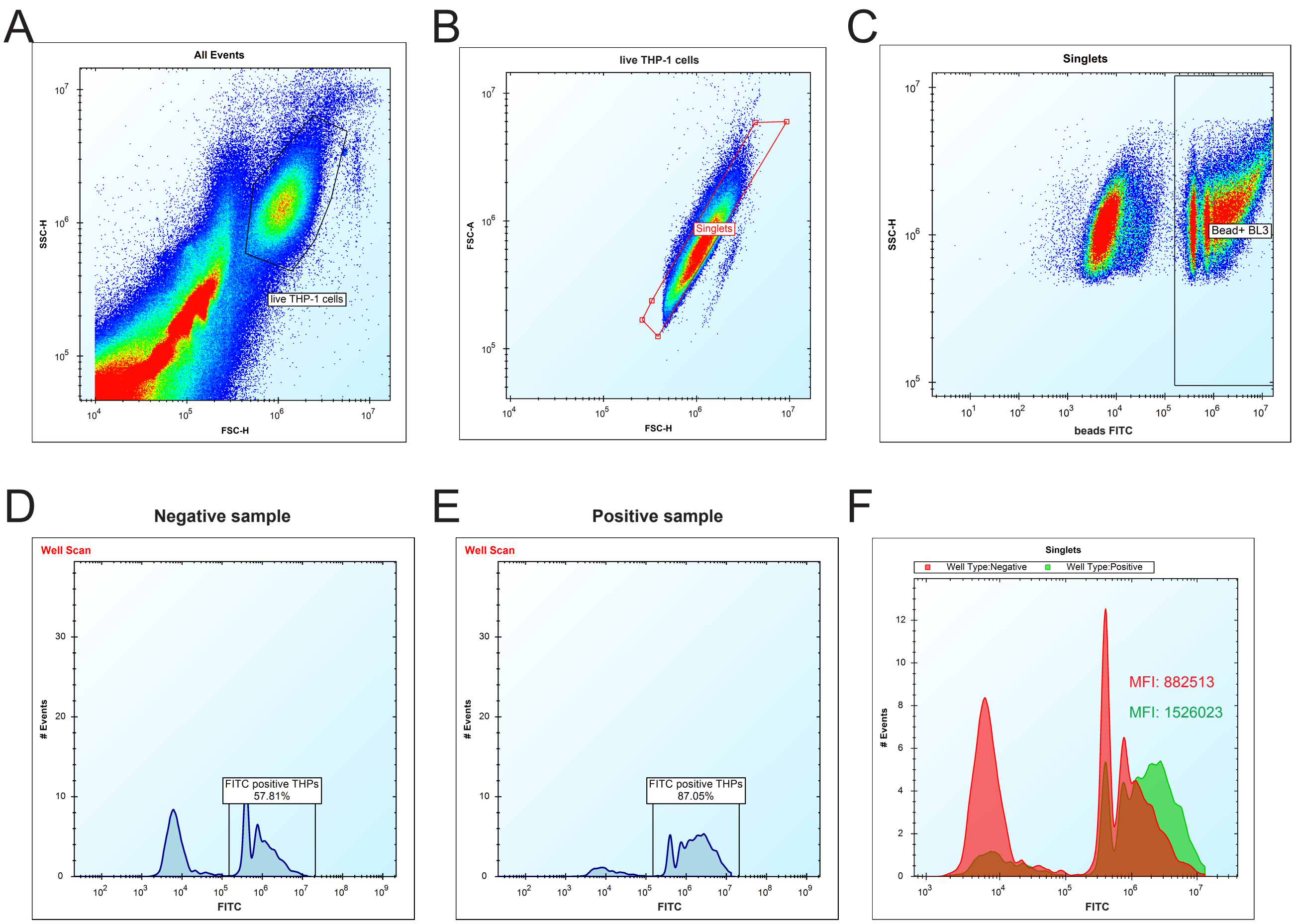


**Supplementary Figure 3. Gating strategy and positive/negative sample for ADMP.** (A) Gating strategy for gating on live THP-1 cells. (B) Gating strategy for single cells. (C) Gating strategy for gating on bead-FITC-positive cells. (D) Example of the percentage of cells positive for beads for a negative sample. (E) Example of percentage of cells positive for beads for a positive sample. (F) Overlay of the fluorescence of cells for a positive sample (green) and negative sample (red). The median fluorescence intensity (MFI) calculated for each sample is shown.

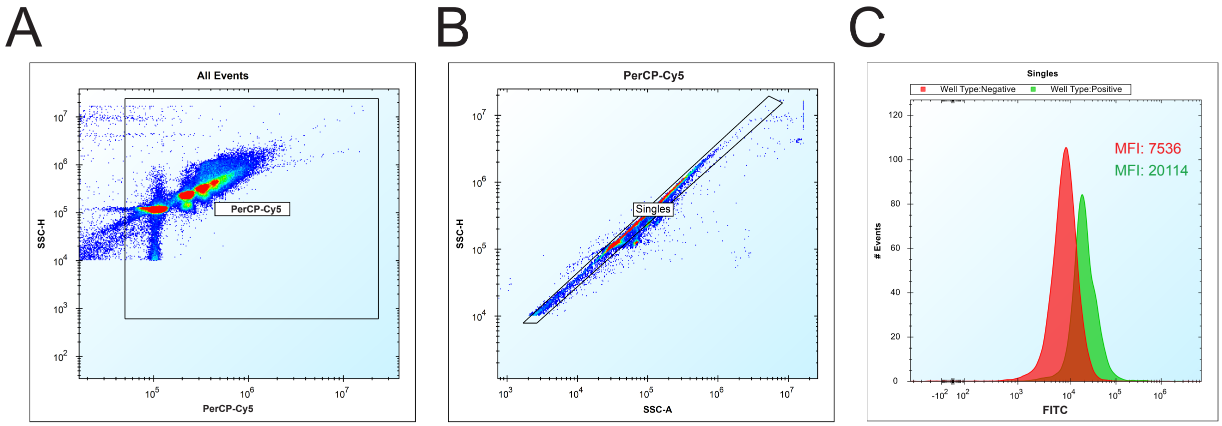


**Supplementary Figure 4. Gating strategy and positive/negative sample for ADCD.** (A) Gating strategy for gating on PerCP-Cy5 fluorescent beads. (B) Gating strategy for single beads. (C) Overlay of the fluorescence of anti-C3, representing the deposition of complement, for a positive sample (green) and negative sample (red). The median fluorescence intensity (MFI) calculated for each sample is shown.
