# Supplementary material for: A single immunization with a modified vaccinia Ankara vectored vaccine producing Sudan virus-like particles protects from lethal infection
Source: NPJ Vaccines. 2022 Jul 25;7:83. doi: 10.1038/s41541-022-00512-x (PMC9314403; doi:10.1038/s41541-022-00512-x)
Supplement: Supplementary file 2 — REPORTING SUMMARY [file 41541_2022_512_MOESM2_ESM.pdf]

nature portfolio

Corresponding author(s): Alexander Bukreyev
Last updated by author(s): Jun 2, 2022

Reporting Summary

Nature Portfolio wishes to improve the reproducibility of the work that we publish. This form provides structure for consistency and transparency in reporting. For further information on Nature Portfolio policies, see our Editorial Policies and the Editorial Policy Checklist.

Statistics

For all statistical analyses, confirm that the following items are present in the figure legend, table legend, main text, or Methods section.

n/a	Confirmed	
☐	☒	The exact sample size (n) for each experimental group/condition, given as a discrete number and unit of measurement
☐	☒	A statement on whether measurements were taken from distinct samples or whether the same sample was measured repeatedly
☐	☒	The statistical test(s) used AND whether they are one- or two-sided<br>*Only common tests should be described solely by name; describe more complex techniques in the Methods section.*
☐	☒	A description of all covariates tested
☐	☒	A description of any assumptions or corrections, such as tests of normality and adjustment for multiple comparisons
☐	☒	A full description of the statistical parameters including central tendency (e.g. means) or other basic estimates (e.g. regression coefficient) AND variation (e.g. standard deviation) or associated estimates of uncertainty (e.g. confidence intervals)
☒	☐	For null hypothesis testing, the test statistic (e.g. F, t, r) with confidence intervals, effect sizes, degrees of freedom and P value noted<br>*Give P values as exact values whenever suitable.*
☒	☐	For Bayesian analysis, information on the choice of priors and Markov chain Monte Carlo settings
☒	☐	For hierarchical and complex designs, identification of the appropriate level for tests and full reporting of outcomes
☒	☐	Estimates of effect sizes (e.g. Cohen's d, Pearson's r), indicating how they were calculated

Our web collection on statistics for biologists contains articles on many of the points above.

Software and code

Policy information about availability of computer code

Data collection	*Provide a description of all commercial, open source and custom code used to collect the data in this study, specifying the version used OR state that no software was used.*
Data analysis	Flow cytometry data were analyzed using FloJo version 10 and Forecyt software (Sartorius). Images were prepared with GraphPad Prism and Illustrator. Statistical analysis was performed with Graphpad Prism version 6.

For manuscripts utilizing custom algorithms or software that are central to the research but not yet described in published literature, software must be made available to editors and reviewers. We strongly encourage code deposition in a community repository (e.g. GitHub). See the Nature Portfolio guidelines for submitting code & software for further information.

Data

Policy information about availability of data

All manuscripts must include a data availability statement. This statement should provide the following information, where applicable:

- Accession codes, unique identifiers, or web links for publicly available datasets
- A description of any restrictions on data availability
- For clinical datasets or third party data, please ensure that the statement adheres to our policy

The datasets generated during and/or analyzed during the current study are available from the corresponding author on reasonable request.

Human research participants

Policy information about studies involving human research participants and Sex and Gender in Research.

Reporting on sex and gender	*Use the terms sex (biological attribute) and gender (shaped by social and cultural circumstances) carefully in order to avoid confusing both terms. Indicate if findings apply to only one sex or gender; describe whether sex and gender were considered in study design whether sex and/or gender was determined based on self-reporting or assigned and methods used. Provide in the source data disaggregated sex and gender data where this information has been collected, and consent has been obtained for sharing of individual-level data; provide overall numbers in this Reporting Summary. Please state if this information has not been collected. Report sex- and gender-based analyses where performed, justify reasons for lack of sex- and gender-based analysis.*
Population characteristics	*Describe the covariate-relevant population characteristics of the human research participants (e.g. age, genotypic information, past and current diagnosis and treatment categories). If you filled out the behavioural & social sciences study design questions and have nothing to add here, write "See above."*
Recruitment	*Describe how participants were recruited. Outline any potential self-selection bias or other biases that may be present and how these are likely to impact results.*
Ethics oversight	*Identify the organization(s) that approved the study protocol.*

Note that full information on the approval of the study protocol must also be provided in the manuscript.

Field-specific reporting

Please select the one below that is the best fit for your research. If you are not sure, read the appropriate sections before making your selection.

☒ Life sciences ☐ Behavioural & social sciences ☐ Ecological, evolutionary & environmental sciences

For a reference copy of the document with all sections, see nature.com/documents/nr-reporting-summary-flat.pdf

Life sciences study design

All studies must disclose on these points even when the disclosure is negative.

Sample size	The number of samples was the minimum number required to obtain scientifically valid results.
Data exclusions	No data were excluded from the analyses.
Replication	All attempts at replication in the stated conditions were successful
Randomization	Allocation of animals to experimental groups was random.
Blinding	There was no blinding.

Reporting for specific materials, systems and methods

We require information from authors about some types of materials, experimental systems and methods used in many studies. Here, indicate whether each material, system or method listed is relevant to your study. If you are not sure if a list item applies to your research, read the appropriate section before selecting a response.

Materials & experimental systems

n/a	Involved in the study	
☐	☒	Antibodies
☐	☒	Eukaryotic cell lines
☒	☐	Palaeontology and archaeology
☐	☒	Animals and other organisms
☒	☐	Clinical data
☒	☐	Dual use research of concern

Methods

n/a	Involved in the study	
☒	☐	ChIP-seq
☐	☒	Flow cytometry
☒	☐	MRI-based neuroimaging

Antibodies

Antibodies used	anti-SUDV GP (IBT cat # 0202-029); anti-EBOV VP40 (IBT cat # 0301-010); AlexaFluor680 goat anti-rabbit IgG (Invitrogen cat # 21109); goat anti-rabbit IgG secondary antibody conjugated to 6 nm gold particles (Aurion cat # 800.011); HRP-conjugated goat anti-guinea pig antibody (Jackson ImmunoResearch cat # 106-035-003); anti guinea pig-Cy5 conjugated antibodies (Jackson ImmunoResearch cat # 706-175-148); goat anti-EBOV serum (gift of Thomas Ksiazek, UTMB); HRP-conjugated bovine anti-goat IgG (Jackson ImmunoResearch, cat # 805-035-180); human mAb BDBV52 (a gift from James Crowe, Vanderbilt) and HRP-conjugated goat anti-human IgG (SeraCare, cat # 5220-0330); anti-CD66b (Pacific Blue, Clone G10F5; Biolegend cat # 305111); anti-guinea pig C3 antibody conjugated to FITC (MP Biomedicals cat # 0855385)
Validation	See manufacturer's website for validation.

Eukaryotic cell lines

Policy information about cell lines and Sex and Gender in Research

Cell line source(s)	Vero E6, DF-1, HEK293 and THP-1 cell lines were obtained from the ATCC.
Authentication	N/A
Mycoplasma contamination	Vero E6 cells were tested for mycoplasma contaminnation by PCR.
Commonly misidentified lines (See ICLAC register)	N/A

Animals and other research organisms

Policy information about studies involving animals; ARRIVE guidelines recommended for reporting animal research, and Sex and Gender in Research

Laboratory animals	Nine-week-old Dunkin-Hartley female guinea pigs
Wild animals	N/A
Reporting on sex	Only female animals were used in the study.
Field-collected samples	N/A
Ethics oversight	Study was approved by UTMB IACUC

Note that full information on the approval of the study protocol must also be provided in the manuscript.

Flow Cytometry

Plots

Confirm that:

- [x] The axis labels state the marker and fluorochrome used (e.g. CD4-FITC).
- [x] The axis scales are clearly visible. Include numbers along axes only for bottom left plot of group (a 'group' is an analysis of identical markers).
- [x] All plots are contour plots with outliers or pseudocolor plots.
- [x] A numerical value for number of cells or percentage (with statistics) is provided.

Methodology

Sample preparation	Peripheral whole blood from human donors was collected into an acid citrate dextrose (ACD) Vacutainer (BD). Ammonium chloride potassium (ACK) lysis buffer was used to lyse red blood cells. White blood cells were counted and resuspended to a final concentration of 5 x 105 cells/ml in complete growth medium for immediate use in the phagocytosis assay. The white blood cells were stained with fluorophore-conjugated antibodies for 15 minutes at room temperature prior to fixation with 4% paraformaldehyde. Fixed samples were analyzed by flow cytometry within 24 hours of fixation.<br><br>THP-1 cells were fixed with 4% paraformaldehyde, and fixed samples were analyzed by flow cytometry within 24 hours of fixation.
Instrument	iQue
Software	Forecyt (Sartorius)

Cell population abundance	A minimum of 10,000 events were captured for each sample.
Gating strategy	All cell populations were first gated on FSC-A and SSC-A. To gate on neutrophils, SSC-A high populations were selected, followed by gating on CD66b positive cells. As no additional markers were used for THP-1 cells, cells were gated by FSC-A and SSC-A only.

☒ Tick this box to confirm that a figure exemplifying the gating strategy is provided in the Supplementary Information.
